# Mastery Exams: Giving Students a Second Opportunity to Demonstrate Understanding

**DOI:** 10.1002/jdd.70075

**Published:** 2025-11-06

**Authors:** Alexandra Rasdal, Eleanor Streitwieser, Cynthia Rabuck, Faizan Alawi

**Affiliations:** ^1^ School of Dental Medicine University of Pennsylvania Philadelphia Pennsylvania USA

**Keywords:** assessment, education, wellness

## Abstract

Various stressors can affect student exam performance, and a single weak result may not reflect true understanding. To address this, a “Mastery” exam policy was introduced in the 2021–2022 academic year. If a student scores below 80% on a didactic or pre‐clinical laboratory exam worth at least 10% of their final course grade, they can choose to retake the exam. The reassessment is a new exam, and may differ in format from the original. The formally recorded grade is the higher of the two scores, up to a maximum of 80%.

**Method:**

Data from the 2021–2024 academic years were analyzed, tracking exam and course grades for all students. Original scores were retained for comparison with final grades before and after Mastery.

**Results:**

Overall, 47.1% of the students completed at least one Mastery exam, with 83.6% improving their scores. At our institution, students are placed on academic probation after one course failure, and risk dismissal after a second failure. Improved performances on Mastery exams resulted in 85.2% of students originally at risk of at least academic probation avoiding any formal administrative action. The initiative was well‐received—89.8% of surveyed students reported improved morale, well‐being, and reduced exam‐related stress.

**Conclusion:**

This institution‐wide initiative yielded several important academic outcomes, including fewer final course failures and fewer students at risk of academic probation or dismissal. This supports the notion that any given exam may be only a reflection of the student's ability to perform to their capabilities at the specific time the exam is administered.

## 1 Introduction

Critical thinking and clinical reasoning are essential to the development and maturation of a dental student. The student must develop the cognitive skills to analyze, evaluate, integrate, and eventually apply much of their classroom learning to patient evaluation, diagnosis, and/or management. This is at least partially achieved through the student's mastery of course content and, in a rigorous and fast‐paced dental school curriculum, often through the use of examinations. This is especially true during the first two years of the curriculum during which many of the foundational biomedical courses are typically presented to the students [1, 2].

While successful exam outcomes are only one objective measure of a student's potential to demonstrate mastery of course content, they remain an effective tool for assessing knowledge [3, 4]. However, academic and exam‐related stress are well‐documented, and the importance placed on outcomes can heighten stress for some students [5, 6, 7]. Depending on an exam's weight, weak performance may negatively impact a final grade, affecting emotional well‐being, confidence, and potentially future academic success. Insufficient preparation may lead to poor exam performance. However, physical and emotional stressors—such as illness, grief, or personal relationship challenges—may also hinder a student's ability to focus during study or apply knowledge during exams [8]. As a result, exam outcomes may not accurately reflect mastery. This may be particularly relevant for first‐year dental students, who are still adjusting to the demands of dental school while adapting to a new environment, social network, and the self‐induced pressure of maintaining past academic success [7, 8].

At the University of Pennsylvania School of Dental Medicine (Penn Dental Medicine [PDM]), students are considered to have sufficiently demonstrated learning by achieving at least 70% (passing grade) on an exam and/or final course grade. However, we want our students to strive for and achieve higher than just passing. In recognition that a relatively weak exam performance may not be indicative of a student's understanding of the course material, a “Mastery” exam policy was implemented in academic year 2021–22. If a student scores below a “B” (80%) on a didactic or pre‐clinical laboratory examination worth at least 10% of their final course grade, they are permitted a second opportunity to take the assessment. The formally recorded grade is the higher of the two scores, up to a maximum of 80%. In general, the reassessment is a new examination, and it may be administered in a different format than the original. The exam is typically administered within two weeks after posting of the original examination grade, and students are not permitted the opportunity to review their original exam prior to taking the Mastery exam.

At PDM, exams in first‐ (D1), second‐ (D2), and third‐ (D3) year courses are administered either in the classroom setting or in the preclinical laboratory; these latter exams are simulation exercises. Fourth‐year (D4) students only complete summative competency assessments including Clinical Competency Examinations and Objective Structured Clinical Examinations, which are not eligible for Mastery. Described herein, we present three full academic years of Mastery exam data across five distinct classes, and only from classroom‐based multiple‐choice exams.

This institution‐wide initiative yielded several important academic outcomes, including a reduction in the total number of final course failures and fewer students at risk of academic probation or potential dismissal from PDM. In addition, the initiative was highly regarded by students, as evidenced by a reported increase in morale and a reduction in self‐reported stress.

## 2 Methods and Materials

### 2.1 Mastery Exam Data Collection

This study was granted an exemption from Institutional Review Board (IRB) review (IRB Protocol Number 854426). Participation in the Mastery exams was voluntary and limited to students in their first through third years who scored below 80% on the original didactic exam. Across the three academic years of study, a total of 1590 students were eligible, of which 749 (47.1%) participated in at least one Mastery exam.

Students were notified of the opportunity to take the Mastery exam through class wide announcements released alongside the original exam results. Students were given a 24‐hour period to register after which eligibility to take the exam expired. However, students were not obligated to take the Mastery exam after they registered.

Following completion of the Mastery exams, grades from both the original and Mastery versions were extracted from ExamSoft (Dallas, TX) and compiled into an Excel spreadsheet (Microsoft 365, Redmond, WA). This allowed for individual‐level tracking of student performance across the academic year and included all exams and courses. In comparing original and Mastery exam scores, we calculated two key metrics: The percentage of cases in which students improved their scores and the frequency with which students achieved scores that met the ≥ 80% threshold.

### 2.2 Final Course Grade Analysis

Electronic grade books embedded within Canvas LMS (Salt Lake City, UT), were reviewed for all courses that offered at least one Mastery exam. A projected final grade in the absence of Mastery was also calculated for each student by substituting their original exam grade in place of the Mastery score, using the data extracted from ExamSoft. All analyses and calculations were conducted in Excel using built‐in statistical and logical functions.

To evaluate the impact of the Mastery exam policy on overall class‐wide exam averages, historical data from ExamSoft were used to compare pre‐policy exam scores (July 2018–June 2021) to post‐policy scores (July 2021–June 2024). Due to curriculum changes during the COVID‐19 pandemic, not all exams remained similar. A representative sample of exams was analyzed, including 15 D1 exams, 11 D2 exams, and 4 D3 exams.

### 2.3 Anonymous Student and Faculty Feedback

A Qualtrics survey was distributed to all predoctoral students (*n* = 689) enrolled within PDM during academic year 2023–2024; we did not exclude any students from participating. A total of 213 students responded (D1 = 45, D2 = 64, D3 = 58, D4 = 48). The survey included 15 questions on various topics including perceived usefulness of Mastery exams, impact on student wellbeing, and administrative concerns.

A separate Qualtrics survey was distributed to all course and module directors; eleven faculty members responded. The survey included 13 questions on topics related to faculty perceptions of the Mastery policy and the potential impact on academic outcomes and students’ wellbeing. Both surveys included an option for additional written comments and feedback.

## 3 Results

At PDM, students are considered to have sufficiently demonstrated learning by scoring at least 70% (passing grade) on an exam and/or final course grade. However, we want our students to strive for and achieve higher than just passing. The Mastery Exam policy, established through the joint efforts of the Dean, department chairs, and other senior administrators, was instituted to afford students an additional opportunity to demonstrate the requisite level of understanding through the use of an alternative examination covering the same content as the original assessment. The policy was drafted and formalized by the Academic Dean and presented to the PDM Faculty Senate for discussion and voting. The Senate overwhelmingly approved the policy for implementation ahead of the 2021–2022 academic year. The written policy was then added to the PDM Student Handbook and explained during orientation for D1, D2, and D3 students.

From 2021–2024, most didactic and preclinical lab courses included two or three exams, with Mastery exams available only to students initially scoring below 80%. In the first year of this initiative, Mastery applied to all exams and quizzes regardless of their contribution to final course grades. Some students chose to take Mastery even when the original assessment (usually a short quiz) was worth less than 3% of the final grade. For example, if a student scored 70% on a quiz (2.1 out of 3 points) and improved to 80% on the Mastery, their final grade would increase by no more than 0.3 points. While this might have reflected a better understanding of the course material, the time spent re‐reviewing content for low‐impact quizzes was later deemed counterproductive. After reviewing cumulative data from the first year, PDM faculty approved a policy change before the 2022–2023 academic year: Only exams worth at least 10% of the final course grade would qualify for Mastery. This change led to 212 fewer Mastery exams administered in 2022–2023 compared to 2021–2022 (Table 1).

**TABLE 1:** Mastery exams across three academic years^a^.

Academic year	2021–2022			2022–2023			2023–2024			Overall
Dental student class^b^	D1	D2	D3	D1	D2	D3	D1	D2	D3	
Total students in each class	148	181	177	152	198	181	168	187	198	1590
Number of unique students who completed Mastery exams (% of total students in class)	89 (60.1%)	102 (56.4%)	72 (40.7%)	99 (65.1%)	105 (53.0%)	56 (30.9%)	76 (45.2%)	81 (43.3%)	69 (34.8%)	749 (47.1%)
Total number of scheduled Mastery exams across all courses^c^	33	36	13	30	27	11	28	24	16	218
Total number of individual Mastery exams administered	407	339	109	304	255	84	257	198	118	2,071
Number of Mastery exams completed by unique student (% of unique students)										
One	26	38	45	41	51	34	27	34	40	336 (44.9%)
Two	19	23	21	21	22	18	10	23	15	172 (23.0%)
Three	8	11	3	5	13	2	14	9	8	73 (9.7%)
Four	3	6	2	11	8	2	8	2	6	48 (6.4%)
Five or more	33	24	1	21	11	0	17	13	0	120 (16.0%)
Mean Mastery exams per unique student (Standard deviation)	4.6	3.3	1.5	3.1	2.4	1.5	3.4	2.4	1.7	2.7 (1.0)

^a^ Only didactic mastery exam data are tabulated herein

^b^ D1 = first year Class, D2 = second year Class, D3 = third year Class

^c^ After the first year of implementation, the Mastery exam policy was modified to limit eligibility to exams worth at least 10% of the final course grade. This resulted in a reduction beginning in academic year 2022–2023 in the overall number of eligible exams across all didactic courses and reduced the total number of Mastery exams administered.

### 3.1 Mastery Exams Administered and Completed by Dental Students, and Associated Exam Grade Outcomes

From academic years 2021–2022 to 2023–2024, there were five distinct classes that completed Mastery exams (Table 1). The graduating class of 2025 is the only group that was eligible for Mastery exams in all three years of the initiative. The graduating classes of 2024 and 2026 had two years of exam data and the classes of 2023 and 2027 had one year of data included in this study. Overall, 47.1% of all D1, D2, and D3 students completed one or more Mastery exams. These exams were not “easier” than the original exams with only 52.9% of all students achieving the 80% grading threshold. However, 83.6% of all students earned higher grades on the Mastery exams compared to the original exams, and with an average grade improvement of 15.2 ± 9.7% (Table 2A). Notably, 67.3% of students who completed only a single Mastery exam in any academic year (44.9% of all students who completed Mastery; Table 1) did achieve the 80% threshold with an average performance that was 16.1 ± 9.4% higher than their original exam grades (Table 2B). In comparing exam grades across multiple courses in the three years before and after implementation of Mastery, there was no statistically significant difference in class averages (*p* > 0.05; Figure S1). Thus, while individual students did benefit from improved exam grades following Mastery within any given course, there was no overall boost in class averages for those exams compared to prior years.

**TABLE 2A:** Mastery exam grade outcomes across three academic years^a^.

Academic year	2021–2022			2022–2023			2023–2024			
Dental student class	D1	D2	D3	D1	D2	D3	D1	D2	D3	Overall
Mastery exams with grade improvement over original exam grade	83.5	84.7	92.7	83.2	84.3	81.0	80.2	85.9	78.0	83.6
Mastery exams with grade ≥ 80%	46.4	58.7	55.0	44.4	59.2	58.3	43.6	66.2	58.5	52.9
Mean % grade improvement (standard deviation)	16.9 (11.0)	17.0 (10.8)	14.9 (7.9)	13.8 (8.7)	15.9 (10.1)	14.5 (7.9)	12.6 (8.5)	14.8 (7.9)	13.5 (8.0)	15.2 (9.7)

^a^ Data reported as % of total.

**TABLE 2B:** Mastery exam grade outcomes for students who completed only one exam within an academic year^a^.

Academic year	2021–2022			2022–2023			2023–2024			
Dental student class	D1	D2	D3	D1	D2	D3	D1	D2	D3	Overall
Total number of students	26	38	45	41	51	34	27	34	40	336
Improved grade after Mastery^a^	92.3	89.5	91.1	82.9	94.1	76.5	85.2	97.1	72.5	86.9
Earned Mastery grade ≥ 80%^a^	80.8	78.9	60.0	46.3	80.4	58.8	55.6	88.2	57.8	67.3
Mean % grade improvement (Standard deviation)	18.4 (10.7)	19.3 (10.1)	15.2 (7.9)	12.6 (8.8)	17.5 (11.9)	18.7 (8.0)	12.4 (7.7)	14.9 (6.0)	15.3 (8.4)	16.1 (9.4)

^a^ Data reported as % of total.

### 3.2 Mastery Exam Outcomes for Students Who Failed Original Exam

The original stated intent of the Mastery initiative was to permit students to re‐assess in the uncommon instances where their studying and associated exam performances were adversely influenced by “life” events or the students experienced an unexpected underperformance with no appreciable explanation. We also acknowledge that at least some students may have underachieved simply because of poor or insufficient exam preparation without external distractors. However, we did not require students to explain why they underperformed, and we did not exclude or deny any eligible students from completing Mastery exams.

Before launching the initiative, we expected that the majority of students would likely take no more than one or two Mastery exams in any given year. This prediction held true as 69.9% of all students did only take one or two of these exams within an academic year (Table 1). Unexpectedly, a small cohort of students, almost exclusively in D1 and D2, found themselves eligible and felt compelled to take five or more Mastery exams (Table 1).

Closer review of the academic records of these latter students revealed that they had consistently lower grades than their peers, including original exam grades that were often barely passing or exam failures (grade < 70%). PDM has a rigorous curriculum and throughout the 3‐year timeframe of this study, 1080 exams originally scored below 70% (Table 3). This does not mean that there would have been a corresponding number of course failures since almost all didactic courses had at least two or more graded summative assessments, often accompanied by other graded activities. Nonetheless, 71.2% of students originally at risk of documenting a failing exam grade earned a passing Mastery exam grade, and 41.7% of these students scored at least 80%. Overall, these students had a grade improvement of 20.2 ± 10.3% relative to their original failing exam grades supporting the notion that even students who earned relatively weak exam grades benefited academically by taking Mastery exams. The counterpoint was that because these same students felt compelled to take multiple Mastery exams, this could have adversely impacted their academic performances in other courses. If the student earned another low grade on the Mastery exam, course directors then offered the student an opportunity to participate in peer tutoring to provide additional learning support.

**TABLE 3:** Mastery exam grade outcomes following original failing exam grade of < 70%^a^.

Academic year	2021–2022			2022–2023			2023–2024			
Dental student class	D1	D2	D3	D1	D2	D3	D1	D2	D3	Overall
Total number of original exams in fail range	261	202	62	171	119	40	119	77	29	1080
Earned Mastery grade ≥ 70%^a^	67.4	76.2	77.4	59.6	80.7	70.0	65.5	87.0	69.0	71.2
Earned Mastery grade ≥ 80%^a^	35.6	52.5	38.7	33.3	53.8	45.0	29.4	54.5	37.9	41.7
Mean % grade improvement (Standard deviation)	18.6 (9.6)	19.1 (8.8)	18.2 (7.7)	17.9 (8.2)	21.8 (10.5)	18.8 (8.1)	17.5 (9.0)	19.1 (8.8)	18.6 (9.6)	20.2 (10.3)

^a^ Data reported as % of total.

### 3.3 Impact of Mastery Exams on Final Course Grades Including Passing Courses

PDM employs letter grading for all courses. The grading scheme for didactic courses is described in Figure S2. Of the 749 students who completed at least one Mastery exam, 62.8% of students earned higher numerical final course grades and 81.8% of students earned higher letter grades than they would have earned without the help of this initiative (Table 4A). Overall, the mean letter grade improvement was 1.4 ± 0.7. This means that at least some students improved their final course grade by more than one letter grade.

**TABLE 4A:** Final course grade outcomes.

Academic year	2021–2022			2022–2023			2023–2024			
Dental student class	D1	D2	D3	D1	D2	D3	D1	D2	D3	Overall
Number of courses with at least one Mastery exam	14	14	10	17	12	10	14	14	11	116
Unique students who took at least one Mastery exam in at least one course	89	102	72	99	105	56	76	81	69	749
Final course grade improved compared to original expected grade (%)	63.0	58.3	72.0	69.9	65.7	53.6	56.9	65.7	51.3	62.8
Students earned higher letter grade in at least 1 course after Mastery (%)	80.9	72.3	77.8	85.9	74.3	60.7	77.6	77.8	65.2	81.8
Mean letter grade improvement (Standard deviation)	1.7 (0.9)	1.5 (0.7)	1.5 (0.7)	1.4 (0.7)	1.3 (0.6)	1.4 (0.7)	1.3 (0.5)	1.2 (0.5)	1.2 (0.5)	1.4 (0.7)

At PDM, a course failure results in placement of the student on academic probation, with risk of dismissal if there is a subsequent course failure. For students originally at potential risk of course failure, 85.2% earned a passing course grade that was directly attributable to earning a higher grade on one or more Mastery exams within the course (Table 4B). Ultimately, 14.8% of “at‐risk” students did fail a course even after completing Mastery exams. However, the total number of students remediating failed courses decreased since launch of the Mastery initiative. In the three academic years preceding Mastery, there were a total of 33 students who were required to undergo formal course remediation. After Mastery was initiated, there were 23 students who failed courses, including only nine students from 2022 to 2024, the majority of whom were D1 students. Therefore, Mastery exams contributed to a reduction in the total number of students placed on academic probation than would have occurred without Mastery.

**TABLE 4B:** Final grade outcomes for students at original risk of course failure.

Academic year	2021–2022			2022–2023			2023–2024			
Dental student class	D1	D2	D3	D1	D2	D3	D1	D2	D3	Overall
Unique students	42	25	11	28	15	7	12	9	6	155
Students who completed course with passing grade	30 (71.4%)	23 (92.0%)	11 (100%)	23 (82.1%)	14 (93.3%)	7 (100%)	11 (91.7%)	9 (100%)	4 (66.7%)	132 (85.2%)
Students who completed course with failing grade^a^	12 (28.6%)	2 (8.0%)	0	5 (17.9%)	1 (6.7%)	0	1 (8.3%)	0	2 (33.3%)	23 (14.8%)

^a^ Students who failed a course were required to remediate during the summer term without an option for Mastery exams. All students successfully completed remediation and matriculated to the next academic year. To date, none of these students have been in academic jeopardy since their original course failure and after they matriculated to the next year.

### 3.4 Mastery Exams Improved Students’ Self‐Reported Wellness and Reduced Exam Stress

In academic year 2023–2024, all students and all faculty included on a course director listserv were surveyed anonymously for their perspectives about the Mastery exam initiative. The complete surveys and results are included as supplemental materials. Students were asked a series of questions that pertained to the number of Mastery exams they completed during their time at PDM, the impact on their final course grades, questions about exam administration, and their perceptions about the impact these exams might have had on their own mental health and stress. Figure 1A highlights selected responses from the survey with at least 138 responses per question. Note that 26.7% of students who responded to the survey acknowledged never having completed a Mastery exam. Most revealing was that 82.6% of all respondents indicated that their personal stress was reduced by simply knowing the option to take a Mastery exam was a possibility. In addition, 89.8% of respondents believed that Mastery exams improved overall student morale and well‐being.

FIGURE 1A: Select student survey questions and responses. Averages may not total 100% due to rounding. B: Select faculty survey questions and responses. Averages may not total 100% due to rounding.

**Figure:**
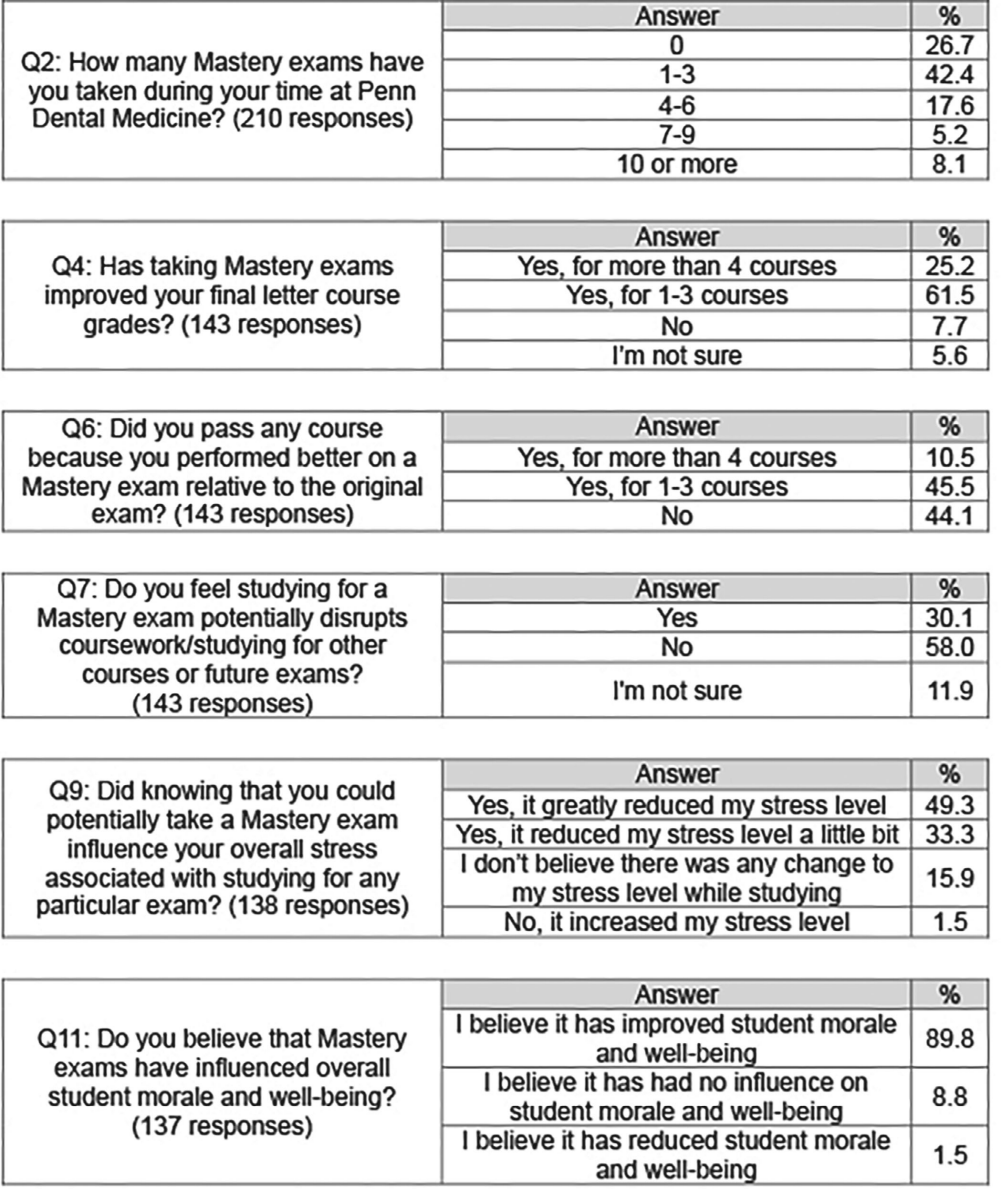

**Figure:**
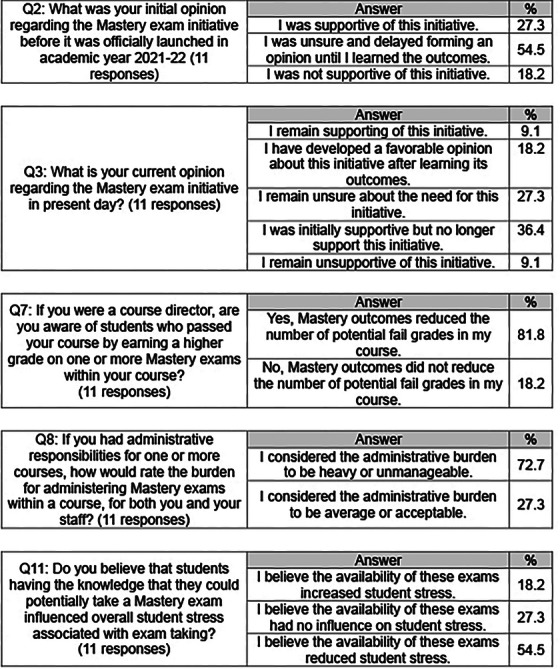

### 3.5 Faculty Had Mixed Opinions About Mastery Exams

Prior to its approval and implementation, the Mastery exam initiative was discussed by the PDM faculty in a school wide forum. During this discussion, there was some doubt expressed about the need for its implementation, and some faculty members believed Mastery exams would actually increase student stress. There were also concerns about unnecessarily coddling the students, grade inflation, and grade equity. For example, some students would earn a B grade (80%) without Mastery whereas others could earn that same grade after taking Mastery. Nonetheless, while all of these concerns had potential merit and were discussed at length, PDM faculty overwhelmingly decided that our students’ academic success and wellness were paramount, and the initiative was approved by a wide margin. With approval of the policy, our faculty acknowledged and accepted the increased effort and resources it would take to create multiple extra exams.

Three years later, the faculty were surveyed for their perspectives about Mastery exams. The questions generally pertained to opinions about Mastery before and after implementation, the impact on final course grades, questions about exam administration, and faculty perceptions about the impact these exams might have had on students’ well‐being. Figure 1B summarizes the responses from selected questions.

Unlike the student survey, there was a very poor response rate by the faculty. However, we did not equate poor survey participation with either apathy or opposition to the Mastery exams; the majority of full‐time PDM faculty had approved implementation of the policy. In addition, some survey recipients were not involved in the original decision‐making.  Nonetheless, while there were too few survey respondents (only 11) to draw meaningful conclusions, the limited data is important to include herein. Most respondents believed there was a heavy administrative burden associated with these exams. Indeed, after implementation, it became clear that there was an increased administrative burden for faculty and staff tasked with designing the exams and proctoring. All exams needed two distinct versions with similar difficulty level, and the time required for in‐person proctoring essentially doubled. While 54.5% of faculty respondents believed that the exams reduced student stress, and despite knowing the positive student objective outcomes achieved by the initiative, faculty opinions about Mastery exams remained mixed, with some faculty in favor and others opposed.

## 4 Discussion

Many dental students are historically high‐achieving students, and oftentimes their emotional well‐being and mental health status, including self‐esteem, are closely tied to their academic performance [6, 7, 8, 9, 10, 11, 12]. Multiple studies have identified impaired mental health as a significant predictor of poor academic performance [8, 10, 11]. From the standpoint of exam preparation and exam taking, impaired mental health should not be limited to depression, anxiety, or other psychiatric illness [8]. There is a plethora of other reasons for which a student's studying or exam performance may be adversely impacted. Apart from insufficient and/or poor exam preparation, other examples might include a poor night's sleep, lingering emotions resulting from the illness or death of a loved one or pet, personal relationship struggles, and/or an emotionally jarring experience immediately preceding an exam, perhaps as otherwise innocuous as being stuck in traffic trying to get to school on time, which may potentially lead to a sense of heightened stress in a susceptible student [8].

In 2023, the Fédération Dentaire International (World Dental Federation) adopted a policy emphasizing the importance of good mental health for not only oral health professionals but also dental students [13]. In addition, dental education institutions were encouraged to develop resources to provide support to students experiencing mental health challenges. PDM and other dental schools have a variety of extracurricular programs to help promote student wellness [8, 9, 10, 11, 12]. However, PDM took the additional approach to implement intracurricular mechanisms to further promote student emotional wellbeing. As just one example, Mastery exams were implemented in recognition that a weak exam outcome may not be indicative of a student's understanding of the course material.

**FIGURE 2:**
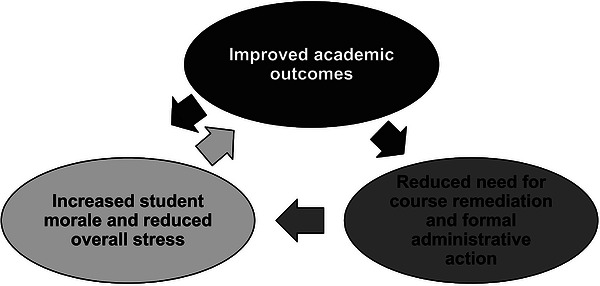
Improved academic outcomes were linked to students’ perceptions of their own morale and stress.

Mastery exams have proven to be very popular with PDM students and have helped improve student morale and self‐reported stress. Students’ academic confidence positively impacts their emotional well‐being, and vice versa (Figure 2) [5, 6, 7, 8]. This was especially true for D1 students who completed Mastery exams more frequently than D2 and D3 students, and who benefited from the availability of these exams as they adjusted to the rigors of the PDM curriculum. Anecdotally, some students acknowledged the Mastery exam policy as being one of the reasons they chose to attend PDM.

At PDM, students are placed on academic probation after one course failure and risk dismissal from the institution after a second failure. Course failure and/or receipt of an administrative letter indicating they have been placed on academic probation may be demoralizing to the affected student, and lead to embarrassment and reduced self‐esteem. Improved performances on Mastery exams resulted in 85.2% of students originally at risk of at least academic probation avoiding any formal administrative action. Moreover, students who failed an original exam scored an average of 20.2 ± 10.3% higher on the Mastery exam; 41.7% of these students scored at least 80% on the re‐assessment (Table 3). This further supports the notion that any given exam may be only a reflection of the student's ability to perform to their capabilities at the specific time the exam is administered. Overall, 81.8% of students who completed at least one Mastery exam within a course earned a higher letter grade in that course.

Prior to implementation of the Mastery exam policy, some faculty members expressed concerns about students potentially undermining their own self‐reliance and ability to acquire and retain the necessary knowledge. However, this concern has not materialized. While Mastery helped a small cohort of students pass courses, these re‐assessments did not mask general academic weaknesses. None of these same students found themselves on the cusp of subsequent course failure or risk of dismissal from PDM for academic reasons. In addition, every objective metric PDM uses to assess our curricular outcomes has been consistently excellent. Throughout the entire 3‐year reporting period of this study, we had 99.8% on‐time graduation, Integrated National Board Dental Examination cumulative d‐scores of +1.22 (Class of 2024) and +1.23 (Class of 2025) with a 99.1% first‐time pass rate (these students had two and three years, respectively, of Mastery opportunity), very low numbers of students who required remediation (as shown in Table 4B), and more than 96% of students who applied to postgraduate training programs were accepted. This reaffirms to us that not only did our students develop the ability to acquire the necessary knowledge for dental practice but that they retained their knowledge throughout their education. While we don't believe Mastery exams are the only reason for our students’ collective learning success, we firmly believe that this initiative did contribute to the overall success of our students.

While Mastery exams became an important component of PDM's assessment strategy, there were some recognized challenges with this initiative:

1. Some weaker students tended to complete a high number of Mastery exams.
2. In a course with three or more exams, eligible students were more likely to take a Mastery exam after the first exam without knowing the impact their original exam grade would have had on their final course outcome. This was particularly true for students who scored between 70% and 79%, and for whom an improved Mastery exam grade retrospectively proved to be unnecessary since it had little to no impact on the final course letter grade.
3. There was a potential adverse impact additional preparation for Mastery exams had for some students studying for other courses running in parallel and whose exams were scheduled often times the same week as the Mastery exams.
4. Due to multiple scheduling constraints, students were often required to miss other scheduled academic activities in order to take a Mastery exam.
5. Although the stated intent was for faculty to create entirely new Mastery exams, on very rare occasion, course directors simply permitted the students to re‐take the original examination. When this was brought to the attention of the Academic Dean, the department chair was informed and corrective measures implemented to ensure this wasn't repeated for future exams.
6. There was an increased administrative burden for faculty and staff associated with the creation of these additional exams and their proctoring. To help mitigate this, the Office of Academic Affairs centralized and streamlined some of the administrative effort associated with these exams. In addition, the department chairpersons collectively agreed to release their administrative staff to help with exam proctoring as needed, even for courses that weren't managed by their departments.

PDM recently launched a new pre‐doctoral curriculum in academic year 2024–2025, which provided an opportunity to further evolve the Mastery exam policy to address some of the aforementioned challenges. While the Mastery exam grade outcomes were mostly similar to the data reported herein, there were 54% fewer Mastery exams administered in D1 and D2 in 2024–2025 compared to 2023–2024, and only three students failed courses under the new curricular structure (not shown). This has helped to markedly reduce concerns from the faculty and staff about the administrative effort associated with Mastery exams.

## 5 Conclusion

PDM has a robust student support program, including several extracurricular faculty‐ and/or peer‐driven student wellness initiatives. Students are eligible for peer tutoring and faculty make themselves available to answer questions. In addition, for students who continue to demonstrate academic challenges, they are referred to the Weingarten Learning Center at the University of Pennsylvania for free consultation with expert academic STEM counselors for guidance related to improving time management skills. Mastery exams were designed to be an intracurricular student and academic wellness initiative but without compromising our academically rigorous educational program. This novel and successful program not only led to positive objective academic and administrative outcomes, it also improved our students’ overall morale and reduced self‐reported stress. Provided that faculty are willing and resources are available, we encourage other dental schools to consider implementing similar types of intracurricular policies that may help students potentially acclimate to the rigors of dental education and reduce the inherent stress associated with assessment and individual exam outcomes.

## Author Contributions

A.R. tabulated and analyzed all data and contributed to writing and review of this manuscript; E.S. oversaw implementation of all Mastery exams, tabulated data and reviewed this manuscript; C.R. contributed to writing and review of this manuscript; F.A. conceptualized and oversaw the entire project, contributed to all aspects of data analysis and wrote the manuscript.

## Conflicts of Interest

The authors declared no conflicts of interest.

## Supporting information




**Figure S1**: Exam grades before and after implementation of Mastery exams.
**Figure S2**: Grading scheme for all PDM didactic courses

## Data Availability

The data that support the findings of this study are available on request from the corresponding author. The data are not publicly available due to privacy or ethical restrictions.
