# Supplementary material for: Mastery Exams: Giving Students a Second Opportunity to Demonstrate Understanding
Source: J Dent Educ. 2025 Nov 6;90(7):969–79. doi: 10.1002/jdd.70075 (PMC13371999; doi:10.1002/jdd.70075)
Supplement: Supplementary file 1 — Figure S1: Exam grades before and after implementation of Mastery exams. Figure S2: Grading scheme for all PDM didactic courses [file JDD-90-969-s001.pdf]

Figure 1: Exam grades before and after implementation of Mastery exams

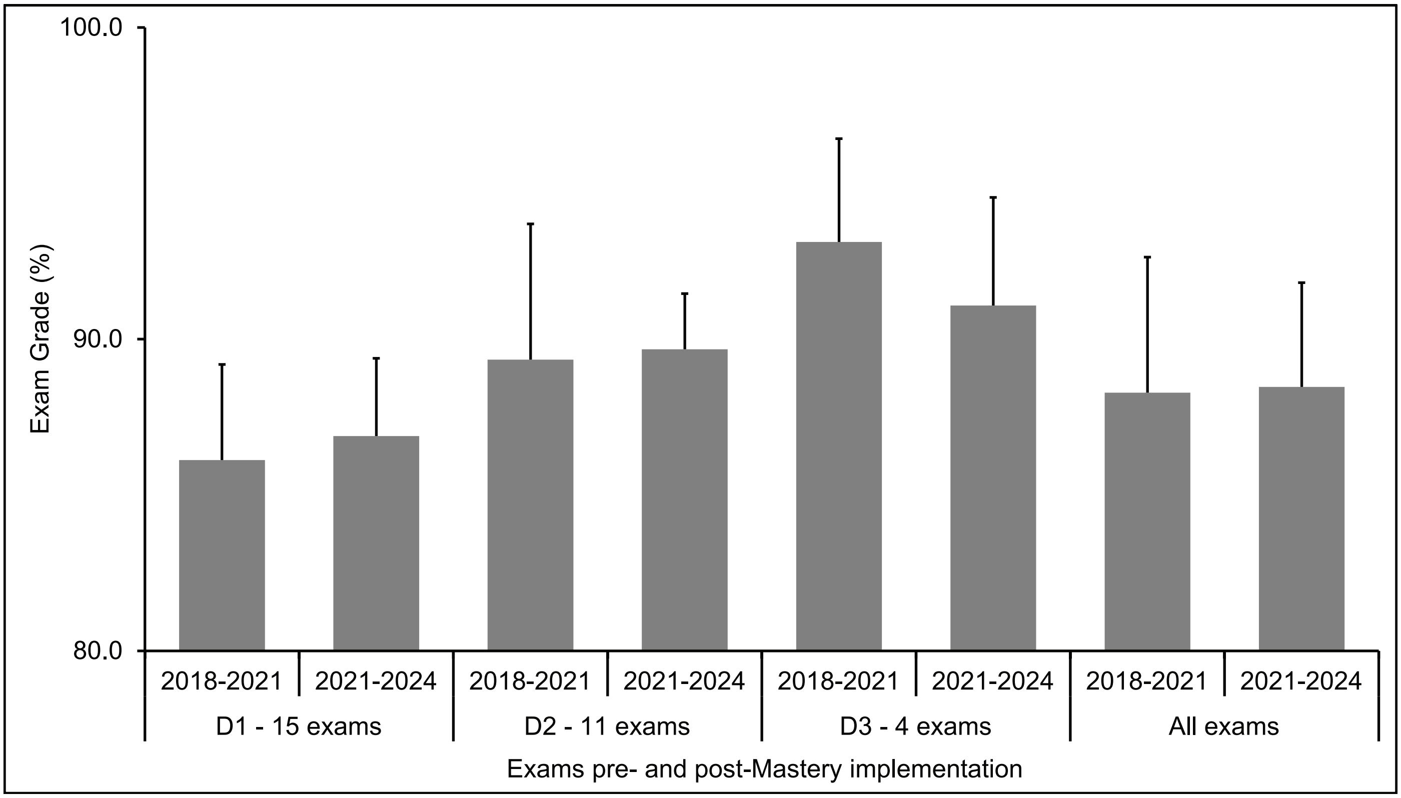

Figure 2: Grading scheme for all PDM didactic courses

COURSE GRADE	EQUIVALENT PERCENT SCORE IN COURSE
H (Honors)	94.5%-100%
A	89.5%-94.49%
B+	85.5%-89.49%
B	79.5%-85.49%
C+	75.5%-79.49%
C	69.5%-75.49%
F (Fail)	Below 69.5%

Mastery Exams

Student Survey

Q1 - Indicate your current class year.

#	Answer	%	Count
1	D1	21.13%	45
2	D2	30.05%	64
3	D3	27.23%	58
4	D4	21.60%	46
	Total	100%	213

Q2 - How many Mastery exams have you taken during your time at Penn Dental Medicine?

#	Answer	%	Count
1	0	26.67%	56
2	1-3	42.38%	89
3	4-6	17.62%	37
4	7-9	5.24%	11
5	10 or more	8.10%	17
	Total	100%	210

Q3 - Have you typically done better or worse in the Mastery exam compared to the original exam? If you have taken multiple Mastery exams, please indicate your overall general performance.

#	Answer	%	Count
1	Usually scored at least 80%	70.83%	102
2	Often better but not always achieved 80% (> 5% grade improvement)	22.92%	33
3	Marginally better (< 5% improvement)	3.47%	5
4	No difference in my grades	0.00%	0
5	Sometimes scored lower	2.78%	4
6	Usually scored lower	0.00%	0
	Total	100%	144

Q4 - Has taking Mastery exams improved your final numerical course grades?

#	Answer	%	Count
1	Yes, for more than 4 courses	28.17%	40
2	Yes, for 1-3 courses	64.08%	91
3	No	4.23%	6
4	I’m not sure	3.52%	5
	Total	100%	142

Q5 - Has taking Mastery exams improved your final letter course grades?

#	Answer	%	Count
1	Yes, for more than 4 courses	25.17%	36
2	Yes, for 1-3 courses	61.54%	88
3	No	7.69%	11
4	I’m not sure	5.59%	8
	Total	100%	143

Q6 - Did you pass any course because you performed better on a Mastery exam relative to the original exam?

#	Answer	%	Count
1	Yes, for more than 4 courses	10.49%	15
2	Yes, for 1-3 courses	45.45%	65
3	No	44.06%	63
	Total	100%	143

Q7 - Do you feel studying for a Mastery exam potentially disrupts coursework/studying for other courses or future exams?

#	Answer	%	Count
1	Yes	30.07%	43
2	No	58.04%	83
3	I'm not sure	11.89%	17
	Total	100%	143

Q8 - Did knowing that you could potentially take Mastery exams influence your overall stress associated with studying for all exams?

#	Answer	%	Count
1	Yes, it greatly reduced my stress level	50.72%	70
2	Yes, it reduced my stress level a little bit	34.78%	48
3	I don’t believe there was any change to my stress level while studying	13.77%	19
4	No, it increased my stress level	0.72%	1
	Total	100%	138

Q9 - Did knowing that you could potentially take a Mastery exam influence your overall stress associated with studying for any particular exam?

#	Answer	%	Count
1	Yes, it greatly reduced my stress level	49.28%	68
2	Yes, it reduced my stress level a little bit	33.33%	46
3	I don’t believe there was any change to my stress level while studying	15.94%	22
4	No, it increased my stress level	1.45%	2
	Total	100%	138

Q10 - Did knowing that Mastery exams were a policy at Penn Dental Medicine influence your decision to enroll within the school?

#	Answer	%	Count
1	Yes, it was one of the reasons I chose to come to PDM	23.36%	32
2	No, it had no influence on my decision to come to PDM	51.82%	71
3	Not applicable since I was already a student when the policy was originally implemented	24.82%	34
	Total	100%	137

Q11 - Do you believe that Mastery exams have influenced overall student morale and well-being?

#	Answer	%	Count
1	I believe it has improved student morale and well-being	89.78%	123
2	I believe it has had no influence on student morale and well-being	8.76%	12
3	I believe it has reduced student morale and well-being	1.46%	2
	Total	100%	137

Q12 - How has the communication been about the details of the Mastery exam?

#	Answer	%	Count
1	Very Poor	1.45%	2
2	Poor	4.35%	6
3	Acceptable	28.99%	40
4	Good	36.23%	50
5	Very good	28.99%	40
	Total	100%	138

Q13 - Who has been communicating with you about the Mastery exam? (Select all that apply)

#	Answer	%	Count
1	Course/Module Director	43.08%	112
2	Course Coordinators	31.92%	83
3	Office of Academic Affairs	24.62%	64
4	Other	0.38%	1
	Total	100%	260

Q14 - In general, Mastery exams are given within two weeks of the original exam. How do you feel about this timing?

#	Answer	%	Count
1	Too soon	0.74%	1
2	Appropriate timing	85.29%	116
3	Too late	11.76%	16
4	Other	2.21%	3
	Total	100%	136

Q15 - If Mastery exams were moved to a different time, which would be your preference?

#	Answer	%	Count
1	End of each course	4.41%	6
2	End of each semester	3.68%	5
3	End of the school year	0.00%	0
4	No change to current timing	91.91%	125
	Total	100%	136
